# ASO Author Reflections: Preventing Nephrotoxicities in Ovarian Cancer Patients Undergoing HIPEC

**DOI:** 10.1245/s10434-023-14268-8

**Published:** 2023-09-11

**Authors:** Rosemary N. Senguttuvan, Thanh H. Dellinger

**Affiliations:** Division of Gynecologic Oncology, Department of Surgery, City of Hope National Medical Center (COH), Duarte, CA USA

## Past

The use of hyperthermic intraperitoneal chemotherapy (HIPEC) in ovarian cancer increased substantially in the United States after publication of the OVHIPEC-1 trial in 2018.^1^ As gynecologic oncologists embrace HIPEC as a new standard of care for ovarian cancer patients, careful planning through a multidisciplinary approach with surgical oncologists, anesthesiologists, and intensivists is required to optimize perioperative and clinical outcomes. It is thus imperative to study and report the management of toxicities and complications of HIPEC among ovarian cancer patients. The incidence of renal toxicities among ovarian cancer patients undergoing HIPEC with cisplatin is high. A recent expert consensus guideline from the Peritoneal Surface Oncology Group International (PSOGI) recommended the routine use of nephroprotection for ovarian cancer patients undergoing HIPEC.^2^ Although nephroprotectants such as mannitol and furosemide are used at various institutions, the most commonly recommended agent for nephroprotection during HIPEC in ovarian cancer is sodium thiosulfate (ST). Demonstrated to be safe in the OVHIPEC-1 trial, ST currently is mandated in the OVHIPEC-2 trial.^3^ Additionally, the KOV-HIPEC-01 trial instituted ST use after an interim analysis demonstrated high renal toxicities.^4^ Despite the demonstrated benefit of ST, the level of evidence supporting its use is low, and use of ST as a nephroprotectant during HIPEC is not currently considered the standard of care in the United States.

## Present

The authors’ single-institution experience of HIPEC with and without ST demonstrated the magnitude of renal morbidity with cisplatin and quantified the mitigation of nephroprotection by ST.^5^ In their prospective pilot trial of 40 ovarian and endometrial cancer patients undergoing cisplatin HIPEC, they demonstrated that the addition of ST resulted in a reduction of renal toxicity from 33 to 0%. None of the patients treated with ST in their study experienced renal toxicities, whereas the patients without ST treatment were affected by grades 2 and 3 acute and chronic kidney injuries. The patients treated with ST had no significant change in postoperative creatinine, whereas postoperative creatinine levels rose by a median of 53% in the patients not treated with ST. The patients without ST use also had a longer time to initiation of chemotherapy than those with ST use (58 vs 41 days, respectively). Despite this, the progression-free survival of the patients undergoing HIPEC with primary interval debulking surgery did not differ between those who had ST and those who did not.

## Future

Nephrotoxicity presents a significant morbidity to ovarian cancer patients undergoing HIPEC with cisplatin. Nephroprotection should thus be mandated and consideration given to adding this to the National Comprehensive Cancer Network (NCCN) guidelines for ovarian cancer HIPEC recommendations. The first U.S. randomized HIPEC trial, GOG 3068-HOTT (NCT05659381), currently is being initiated across numerous U.S. sites, and nephroprotection with ST will be strongly recommended, although not mandated. The recent approval of ST (PEDMARK) by the U.S. Food and Drug Administration (FDA) for use with pediatric patients who have localized, non-metastatic solid tumors has led to cost increases for ST, which may have an impact on its use during HIPEC. Fortunately, generic ST remains available at acceptable prices.

The optimal delivery of HIPEC requires careful multidisciplinary planning to enhance patient tolerance and effect the best patient outcomes. Because HIPEC presents a multi-faceted, complex procedure that results in survival benefits for ovarian cancer patients, optimal perioperative management, including premedication, postoperative care, and fluid management, should be further studied and formal practice guidelines established.
